# Essential Oils Reduce Grey Mould Rot of Apples and Modify the Fruit Microbiome during Postharvest Storage

**DOI:** 10.3390/jof9010022

**Published:** 2022-12-22

**Authors:** Giada Schiavon, Marco Garello, Simona Prencipe, Giovanna Roberta Meloni, Fabio Buonsenso, Davide Spadaro

**Affiliations:** 1 Department of Agricultural, Forestry and Food Sciences (DISAFA), University of Turin, Largo Paolo Braccini 2, 10095 Grugliasco, Italy; 2 Centre of Competence for the Innovation in the Agro-Environmental Sector-AGROINNOVA, University of Turin, Largo Paolo Braccini 2, 10095 Grugliasco, Italy

**Keywords:** natural antifungal compounds, biofumigation, *Botrytis cinerea*, metabarcoding, postharvest disease, *Malus x domestica*, SPME-GC-MS

## Abstract

*Botrytis cinerea* is the causal agent of grey mould rot of apples. The efficacy of biofumigation with thyme (*Thymus vulgaris*), savoury (*Satureja montana*), and basil (*Ocimum basilicum*) essential oils (EOs) at 1%, 0.5%, and 0.1% concentrations were tested against *B. cinerea*. In vitro, the results showed 100% growth inhibition at 1% concentration for all oils. Subsequent biofumigation experiments on apples of cultivar ‘Opal’ with 1% EOs showed that, after 60 d storage, thyme and savoury EOs significantly reduced grey mould rot incidence (average incidence 2% for both treatments) compared to the control (7%). Analyses of quality indicated slightly higher fruit firmness for 1% thyme at 30 d and slightly higher titratable acidity for 1% thyme and savoury at 60 d. Sampling of the atmosphere inside the cabinets was performed to characterize and quantify the volatile components of EOs released through biofumigation. Though thymol and *p*-cymene were the main components of thyme EO, the antimicrobial activity was mainly due to the presence of thymol and, to a lower extent, of carvacrol. In savoury EO, carvacrol and *p*-cymene were the main components, whereas in basil EO, linalool and estragole were mainly present. Metabarcoding analyses showed that the epiphytic microbiome had higher richness and evenness compared to their endophytic counterpart. By the end of shelf-life, treatments with thyme EO reduced *B. cinerea* abundance compared to the inoculated control for both endophytes (from 36.5% to 1.5%) and epiphytes (from 7.0% to 0.7%), while favouring a significant increase in *Penicillium* species both in endophytes (from 0.2% to 21.5%) and epiphytes (from 0.5% to 18.6%). Results indicate that thyme EO (1%) and savoury EO (1%) are equally effective in hampering grey mould rot development in vivo.

## 1. Introduction

Apples (*Malus x domestica* Borkhausen), rich in vitamins, fibres, minerals, and polyphenols [1,2], are among the most-consumed fruits worldwide. Apples are available year-round and they are stored for several months after harvest. During storage, apples are susceptible to postharvest pathogens [3]. Controlled conditions and chemical treatments are the most common strategies used to manage diseases and spoilage in apples after harvest [1,4].

Grey mould rot, caused by the fungus *Botrytis cinerea* Persoon: Fries, is one of the diseases that can appear during the postharvest storage of apples. Postharvest diseases have been mainly controlled with chemicals, but nowadays consumers are demanding less chemical pesticide residues in food. Moreover, the legislation, in particular in the European Union, is pushing towards a dramatic reduction of pesticide use and a substitution with more environmentally friendly treatments [5]. Alternative methods to control postharvest diseases could be based on natural products, such as essential oils [6,7,8].

Plant essential oils are biologically active substances and potentially effective as biopesticides for disease management. Many essential oils (EOs) proved their effectiveness against grey mould and brown rot (*Monilinia* species) on pome and stone fruit, strawberries, and table grapes [8,9,10,11]. On apples, various studies have reported the efficacy of EOs against blue mould (*Penicillium expansum*) [12,13,14], grey mould [8,12,15,16], and bitter rot (*Colletotrichum* species) [17,18], as well as *Alternaria* and *Fusarium* rots [17,18,19]. Different researchers described the volatile organic compounds (VOCs) present in EOs, such as thymol, eugenol, *p*-cymene, and carvacrol, which show strong antifungal activity and could be used for grey mould control on fruit [20]. The use of alternative control strategies, such as essential oils, is slowly reaching practical application [21,22].

During plant growth and after harvest, microorganisms play an important role and strictly collaborate with their host. High microbiome diversity is valuable for fruit and vegetables and pathogenic fungi can disturb the microbial balance, by creating a dysbiosis state [23,24]. Microorganisms are an intrinsic element of the horticultural products found as endophytes within tissues and as epiphytes on the surface. Most of these microbes are not pathogens. Their ecology, colonization, activity in fruit quality, fitness, and disease protection are generally unexplained [25]. Microbial community dynamics can change due to fruit physiology and other abiotic factors, including storage, and postharvest treatments [26]. Variations of microorganisms on the peel are linked to fruit decay during storage [23,27,28].

This study aimed to test the efficacy of three EOs during storage (*Thymus vulgaris*, *Satureja montana*, and *Ocimum basilicum* subspecies *basilicum*) and a thymol formulation against *B. cinerea* on apples, both in vitro and in vivo. To verify the persistence of EOs during storage, we characterized the volatile components of EOs during two months of storage in cabinets under controlled conditions by using Solid Phase Micro-Extraction and Gas Chromatography combined with Mass Spectrometry (SPME-GC-MS). We also determined the effect of a postharvest application of thyme EO on the composition of the epiphytic and endophytic fungal microbiota of apples during cold storage.

## 2. Materials and Methods

### 2.1. Essential Oils Preparation

The EOs of thyme (*T. vulgaris*), savoury (*S. montana*), and basil (*O. basilicum* subspecies *basilicum*) used in the assays were purchased from Pranarôm Aromaterapia Scientifica (Ghislenghien, Belgium). An experimental thymol-based formulation, supplied by Xeda International (Saint-Andiol, France), was also tested.

### 2.2. In Vitro Biofumigation Test

For the in vitro test, EOs of thyme, basil, savoury, and the thymol formulation were used at the concentrations of 1%, 0.5%, and 0.1% to evaluate the growth inhibition of two strains of *B. cinerea* isolated from apples (BOT1 and BOT2), taken from the collection of the University of Torino (DISAFA). Potato Dextrose Agar medium (PDA, EMD Millipore Corporation, Darmstadt, Germany) was poured into the Petri dish (15 ml per Petri dish) and the percentage of EO tested (1, 0.5 or 0.1% *v*/*v*) was added after autoclaving. At the same time, Petri dishes with only PDA medium were inoculated with one mycelium plug (8 mm diameter) taken from cultures grown on PDA for 5 days at room temperature (24 ± 1 °C) for both strains of *B. cinerea*. Petri dishes with the EOs were placed on the top of the inoculated Petri dishes with a face-to-face orientation to build a sandwich, and subsequently closed with parafilm. Petri dishes were incubated at 20 ± 1 °C for 5 days, and then the diameter of the pathogen mycelium was measured. Controls were set up using a PDA Petri dish on top of inoculated Petri dishes. The assay was performed twice, each time with three biological replicates.

### 2.3. Efficacy of EOs against Grey Mould Rots on Apples

Scab-resistant ‘Opal’ apples were used and harvested in Saluzzo (CN), Italy. The tests were set up using the three EOs and the thymol formulation. The preparation of EO diffusors was carried out by adding the EOs (1% *v*/*v*) in sterile deionized water (98% *v*/*v*) and Tween 20 (1% *v*/*v*). The oil and medium suspensions were then poured into Petri dishes (15 ml per Petri dish). For each of the four biofumigation treatments, three replicates of 30 apples were set up. Chemical control was inoculated with *B. cinerea* and treated with the fungicide pyrimethanil (Scala^®^, 36.8%, BASF) at 0.067% concentration. Fungicide solution was sprayed on the apples, which were then left to dry before subsequent treatments. Control fruits were inoculated with *B. cinerea*. A healthy control was included, made of healthy fruits. Pathogen inoculation was performed by immersion in a conidial suspension of *B. cinerea* of 1 × 10^4^ cells/mL. For inoculum preparation, two strains of *B. cinerea* (BOT1 and BOT2) taken from the collection of the University of Torino (DISAFA), were cultured on PDA supplemented with 0.0025 g streptomycin (Merck, Darmstadt, Germany) for 30 days with 12 hours photoperiod. Conidia were collected by pouring a 1% suspension of the surfactant Tween-20 and by scraping it with a Drigalsky spatula. Counting was performed by microscopy using Burker’s chamber. Before inoculation, 20% of the fruits were wounded using a sterile tip to promote pathogen infection. Apples were inoculated by immersion for 3 minutes in the conidial suspension. The fruits were allowed to dry for 1 hour before being placed in the cabinets. Plastic boxes with apples were placed in cabinets with six slow-released diffusors. The diffusors were open inside the cabinets and positioned around the boxes. The fruits were stored for a period of 60 days at 1 ± 1 °C and 95% relative humidity and subsequently kept in shelf-life conditions at 23 ± 1 °C for 10 days. The evaluation of disease incidence was carried out after 30 and 60 days of storage and then after 10 days of shelf-life.

Quality analysis, firmness, total soluble solids (TSS), and titratable acidity were measured on three replicates of five apples for each treatment at harvest, after 30 days of storage, and after 60 days of storage. Apples for quality analysis were not inoculated. Firmness was determined using the penetrometer 53200 (Turoni, Italy) with an 11 mm diameter tip that returns a measurement in N/cm^2^. Firmness was measured on two opposite sides of the apple after removing the skin and then averaged. For total soluble solids, the NR151 refractometer (Rose Scientific Ltd., Edmonton, Canada) was used. Values obtained from the measurement are expressed as total soluble solids content (%) for each apple and subsequently averaged for each replicate. The titratable acidity was determined using the extracted and filtered juice. The juice sample was titrated with a 0.1 M NaOH solution until the neutralization reaction at pH 8.2 using the Five Easy Plus pH meter FP20-Std-Kit (Mettler Toledo, Italy). The total acidity value was calculated using the formula:

(V_NaOH_ × 0.0067 × 100)/6, (1)

where 0.0067 indicates the acidity factor of malic acid and 6 indicatesnthe grams of juice used for the titrating solution.

### 2.4. Characterization of Volatile Compounds of EOs in the Cabinets during Storage

Composition of thyme, savoury, and basil essential oils and of thymol formulation was obtained by GC-MS. Characterization of the VOCs of the atmosphere of the cabinets during the storage trial on apples ‘Opal’ was obtained by SPME-GC-MS analysis. The air of the cabinets was sampled at 1, 30, and 60 days of storage. Pure EOs were diluted in vials to 1% in n-hexane (thyme and savoury) or methanol (basil) and injected by direct injection into GC-MS using the split mode (80%). Xedatim was diluted in vials to 0.2% in methanol (WVR, Radnor, PA, USA). Sampling in each cabinet was performed using an SPME fibre assembly polydimethylsiloxane (PDMS) of 30 μm (Supelco Analytical, Bellefonte, PA, USA) for 15 minutes at 1, 30, and 60 days of fruit incubation.

Compositional analyses of EOs were performed using a Shimadzu (Kyoto, Japan) GC-2010 Plus gas chromatography coupled to a GCMS-QP2010 Ultra mass spectrometer (Shimadzu, Kyoto, Japan) and a split-splitless injector (Shimadzu, Kyoto, Japan). The gas chromatograph was equipped with a Zebron ZB-5MSi (Phenomenex, Torrance, CA, USA) fused silica capillary column (30 m × 0.25 mm LxID) with 0.25 µm film thickness. Helium was used as the carrier gas using a linear velocity of 37 cm/s with a constant flow rate of 1.0 mL/min. The pressure was 55 kPa and the total flow was 105 mL/min. The spectra of ion-electron impact at 70 eV were recorded in scan mode (30–700 m/z). For savoury EO, the oven program was set with an initial temperature of 50°C and held for 3 minutes, and the heating ramp for the chromatographic separation was set as follows: 50 °C to 70 °C at a speed of 5 °C/min, isothermal for 5 min, from 70 °C to 90 °C with an increment of 1 °C/min, from 90 °C to 150 °C with heating of 5 °C/min, and, finally, up to 270 °C by heating at 40 °C/min and maintained for 5 min. For thyme EO, the same protocol was used without the isothermal phase at 70 °C for 5 min. Chromatographic separation of the components of the basil EO and of the thymol formulation was conducted following a previously established method [29]. The temperature program was as follows: initial temperature of 40 °C for 2 min, increased by 10 °C/min up to 300 °C, which was maintained for 2 min. Desorption of the compounds from the fibre was conducted in the GC injector at the temperature of 250 °C, and the ion source and interface were fixed at 280 °C.

The relative composition of volatile compounds was calculated by comparing the area of each peak with the area of the total chromatogram. Absolute quantification was calculated for thymol, carvacrol, and estragole by the external calibration method, using a standard calibration curve prepared between 1 and 50 ppm (mg/kg) for each compound. The relative quantification for each VOC was determined using the thymol standard calibration curve for thyme EO and thymol formulation, the carvacrol standard calibration curve for savoury EO, and the estragole calibration curve for basil EO.

### 2.5. Apple Microbiome Sampling

Apples for microbiome sampling were collected three times: after harvest, after 60 days of storage at 1 ± 1 °C, and after 10 days at 23 ± 1 °C (shelf-life). At each sampling, five biological replicates were collected, each one consisting of 15 apples. Samples were collected from a healthy control, inoculated control, fruits treated with pyrimethanil, and fruits treated with thyme EO.

For epiphyte sampling, the surface of the apples was rubbed with sterile cotton swabs, and the tip was deposited in a falcon tube with 8 ml of phosphate-buffered saline (PBS) solution (Merck). The falcon was shaken at 250 rpm for 20 minutes at room temperature, sonicated for 5 minutes at a frequency of 40 kHz and the tips of the cotton swab were squeezed with sterile tweezers and placed in a second falcon, with 4 ml of PBS solution. The falcon was shaken manually, then the tips were squeezed again, and the contents of the two falcons were combined. Falcons were centrifuged at maximum speed for 30 minutes and the supernatant was removed. The pellet was suspended with 2 ml of PBS solution and the liquid was transferred to a new Eppendorf tube. The Eppendorf tube was centrifuged for 30 minutes at maximum speed, the supernatant was removed, and the samples were stored at −20 °C.

For endophyte sampling, apples were washed in 5% bleach for two minutes, then in water for two minutes, and finally with water for two more minutes. The surface of the apples was rubbed with ethanol-soaked cotton, and the fruits were allowed to dry on sterile paper. A 1-cm wide section of peel was cut all around the equator of each fruit and placed in a sterile plastic jar. Samples were placed in liquid nitrogen immediately after sampling and then stored at −20 °C.

### 2.6. Genomic DNA Extraction and Sequencing

The extraction of epiphyte samples was performed with Wizard Genomic DNA Purification Kit (Promega Biotech AB, Finnboda Varvsväg, Sweden), following the protocol suggested by the producers. The endophyte samples were lyophilized, then the DNA was extracted using the kit DNeasy Power Soil (Qiagen, Hilden, Germany), following the manufacturer protocol with minor modifications. Briefly, 0.10–0.15 g of lyophilized sample and 60 µL of C1 solution were added into PowerBead tubes, which were vortexed. The tubes were agitated via Tissue Lyser (Qiagen, Germany) for 30 minutes at maximum frequency and centrifuged at maximum speed for 2 minutes. The supernatant was transferred to a 2 mL tube. Next, 500 µL of lysis buffer SL2 (Macherey-Nagel GmbH & Co. KG, Düren, Germany) was added to PowerBead tubes, which were then vortexed. The tubes were shaken via Tissue Lyser for 30 minutes at maximum frequency and centrifuged at maximum speed for 2 minutes. The supernatant was added to the previously obtained supernatant.

Library preparation, pooling, and sequencing were performed at the Genomics and Microbiome Core Facility (GMCF) of Rush University (Chicago, IL, USA). Amplicons were generated using a two-stage PCR amplification protocol [30]. Briefly, genomic DNA was amplified with primers ITS3-KY02 (GATGAAGAACGYAGYRAA) and ITS4 (TCCTCCGCTTATTGATATGC) targeting fungal ITS2 regions [31]. The primers contained 5’ common sequence tags (known as common sequence 1 and 2, CS1 and CS2; ACACTGACGACATGGTTCTACA and TACGGTAGCAGAGACTTGGTCT, respectively). First-stage PCR amplifications were performed in 10 μl reactions in 96-well plates, using repliQa HiFi ToughMix (Quantabio, Qiagen, Beverly, MA, USA). Primer concentrations were 300 nM. A blocking oligonucleotide (ATTGATATGCTTAAATTCAGCGGGTAACCCCGCCTGACCTGGGGTCGCGTT-C3 spacer [31]) was added to the master mix at a concentration of 1 µM to reduce amplification of the plant host DNA. Thermal cycler conditions were 98 °C for 2 min, followed by 28 cycles of 98 °C for 10 s, 78 °C for 1 s, 55 °C for 1 s and 68 °C for 1 s. Subsequently, a second PCR amplification was performed in 10 μl reactions in 96-well plates using repliQa HiFi ToughMix. Each well received a separate primer pair with a unique 10-base barcode, obtained from the Access Array Barcode Library for Illumina (Fluidigm, San Francisco, CA, USA). One microliter of PCR product from the first stage amplification was used as the template for the second stage, without clean-up. Cycling conditions were 98 °C for 2 min, followed by 8 cycles of 98 °C for 10 s, 60 °C for 1 s and 68 °C for 1 s. Libraries were then pooled and sequenced with a 15% phiX spike-in on a MiSeq V3 flow cell (2 × 300 paired-end reads).

### 2.7. Bioinformatics

Sequence analysis was performed using the QIIME2 suite [32] and custom python scripts. Adapter contamination was removed with the Cutadapt plugin [33], then forward and reverse reads were trimmed to 230 and 200 bases, respectively, based on quality profiles and overlap constraints. Read merging, ASVs generation, and chimera filtering were performed using DADA2 [34]. A Naive-Bayes predictor was trained on the UNITE 8.3 global database [35], integrated with other ITS sequences from the NCBI nucleotide database [36] and used to classify the previously generated ASVs. Reads not belonging to fungi and reads that present a global abundance of less than 0.05% of total sequences and reads that appeared in less than four replications were removed.

The normalization of samples for alpha and beta diversity analyses was performed using the scaling with the ranked subsampling (SRS) approach implemented in the module by the same name [37], with a normalisation value of 9753. The Shannon diversity index and number of observed features were selected as alpha diversity metrics, while the Bray-Curtis dissimilarity index was chosen as the beta diversity metric. Statistical analyses of alpha diversity results were performed using the non-parametric Kruskal-Wallis test, with alpha <0.05. Beta diversity results were used to carry out a Principal Coordinates Analysis (PCoA) with the provided PcoA plugin [38], and a Permutational Analysis of Variance (PERMANOVA) with the Adonis plugin [39,40].

Compositional analyses were performed using custom python scripts. ASVs absolute frequencies were collapsed at the genus level for each sample and converted to relative frequencies, then samples were grouped based on time point, tissue, and treatment. To improve data readability, genera with less than 1% frequency across all groups were collapsed into the “other” category. Finally, data were plotted as histograms.

### 2.8. Statistical Analysis

Statistical analysis was performed using IBM SPSS software version 27.0 (SPSS Inc., Chicago, IL, USA). Data obtained in all experiments were analysed by analysis of variance (ANOVA). The results of the treatments on in vitro inhibition, in vivo efficacy, and on fruit quality analyses were separated at the level of 5% significance level (*p* < 0.005) using Tukey’s test.

## 3. Results

### 3.1. EOs Efficacy Tests against Botrytis Cinerea

The results obtained from the in vitro test showed 100% growth inhibition for both strains of *B. cinerea* tested and for all treatments using 1% concentration, except the strain BOT1 treated with a thymol formulation (96.9% growth inhibition). Both thyme and savoury EOs at 0.5% completely inhibited the fungal growth. EOs at 0.1% concentration resulted in reduced growth inhibition (88.7–89.5% for thyme EO, 78.4–81.7% for savoury EO, 0.9–14.9% for basil EO and 25.6–31.7% for the thymol formulation) compared to the other considered concentrations, and for this reason, were dropped from the subsequent analyses (Table 1).

### 3.2. Efficacy of EOs against Grey Mould Rot on Apples

**Figure 1:**
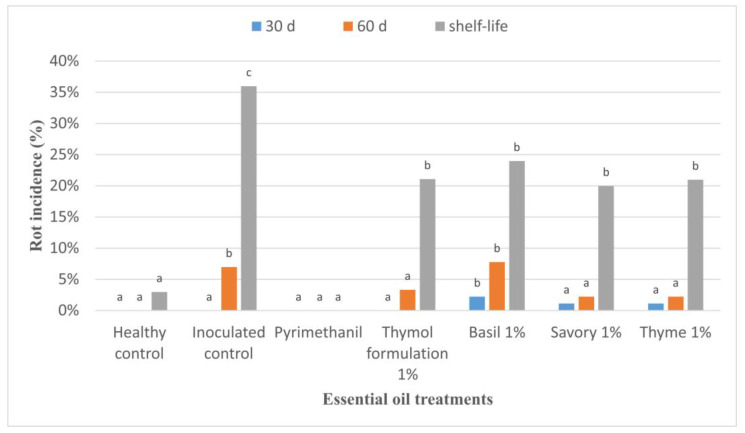
Grey mould incidence on apples ‘Opal’ treated with essential oil biofumigation and stored at 1 ± 1 °C and 95% relative humidity for 60 days and kept in shelf-life at 23 ± 1 °C for 10 days. Values at the same time point, followed by the same letter, are not statistically different by Tukey’s test (*p* < 0.05).

The EOs efficacy tests against grey mould on apples showed the efficacy of the EOs of thyme, savoury, and of a thymol formulation in reducing grey mould rots caused by *B. cinerea* (Figure 1). After 30 days of storage at 1 ± 1°C, no observable rot developed in the apples treated with the thymol formulation at 1%. The incidence of rot in the basil treatment was 2%, and in the savoury and thyme EOs treatments it was 1%. After 60 days of storage at 1 ± 1 °C, the treatments with thyme at 1% and savoury at 1% were statistically different from the inoculated control (*p* < 0.05) showing efficacy in the control of rot development. Rot reduction was 42% for the thyme EO at 1% treatment and 44% for the savoury EO at 1% treatment.

No statistical difference was observed when comparing the incidence of rots for the basil EO at 1% and thymol formulation at 1% treatments to the inoculated control (*p* > 0.05), showing inefficacy in the control of grey mould.

### 3.3. Quality Analyses on Fruits

Quality parameters (firmness, total soluble solids, and titratable acidity) were analysed on apples immediately after harvest, as well as after 30 and 60 days of storage. Analysing the data obtained from ‘Opal’ apples treated with EOs at 1%, no statistically significant difference was observed for the values of total soluble solids, and titratable acidity (*p* > 0.05) after 30 of storage at 1 ± 1 °C compared to the values obtained for untreated apples (Table 2). A statistically significant difference is observed for apples treated with 1% thyme EO, as the latter are found to be firmer than the control after 30 days of storage at 1 ± 1 °C. No statistical difference was observed for the values of firmness, and total soluble solids after 60 of storage at 1 ± 1 °C compared to the values obtained for untreated apples (Table 2). A statistically significant difference is observed for apples treated with 1% thyme and 1% savoury EOs, compared to the values obtained for the control after 60 of storage at 1 ± 1 °C.

### 3.4. Chemical Composition of Essential Oils and Characterization of the Storage Atmosphere

#### 3.4.1. Characterization of Thymol Formulation

The characterization of the atmosphere of the cabinet containing the thymol formulation showed that thymol, the main component of the formulation, was the only volatile organic compound (VOC) present (Table 3). Except for propylene glycol, the other components (74.31%) have a high boiling point and consequently low volatility. The thymol concentration was 4.02 ppm on the first day of storage and decreased to 0.64 ppm at 60 days of storage.

#### 3.4.2. Characterization of Thyme Essential Oil (1%)

Air sampling with SPME fibres in the cabinet during storage, where 1% thyme EO was used, showed that the three most abundant VOCs were thymol, the most abundant component of thyme EO (Table 4), followed by *p*-cymene and terpinene. The values of the three compounds decreased during storage (Table 4): thymol passed from 13.38 ppm at 1 day to 3.85 ppm at 60 days, and during the same period *p*-cymene moved from 11.4 ppm to 2.61 ppm and terpinene from 6.16 ppm to 0.38 ppm.

#### 3.4.3. Characterization of Savoury Essential Oil (1%)

Gas chromatography coupled with mass spectrometry of savoury EO showed that the most abundant VOCs were carvacrol, the main component of savoury EO, followed by *p*-cymene and γ-terpinene. The concentrations of these three compounds in the cabinet atmosphere decreased during storage (Table 5): carvacrol passed from 8.64 ppm at day 1, to 3.34 ppm at day 60. At 30 days, *p*-cymene showed an increase and then passed from 4.69 ppm (day 1), to 25.04 ppm (day 30), it then decreased to 3.95 ppm at the end of storage and finally, γ-terpinene passed from 5.34 ppm at day 1 to 0.90 ppm at day 60.

#### 3.4.4. Characterization of Basil Essential Oil (1%)

From the SPME-GC-MS analysis of the atmosphere of the cabinets, when apples were treated with basil EO, the major VOCs released were estragole (the main component of the basil EO), followed by linalool and α-farnesene (Table 6). Estragole moved from 128.60 ppm at day 1, to 31.42 ppm at the end of storage. Similarly, linalool moved from 57.76 ppm at day 1, to 0.66 ppm at day 60. On the contrary, α-farnesene increased over time, with a peak at 40 days: it moved from 24.68 ppm at day 1, to 111.44 ppm at day 30, and 31.15 ppm at the end of storage.

### 3.5. Microbial Diversity and Composition

**Figure 2:**
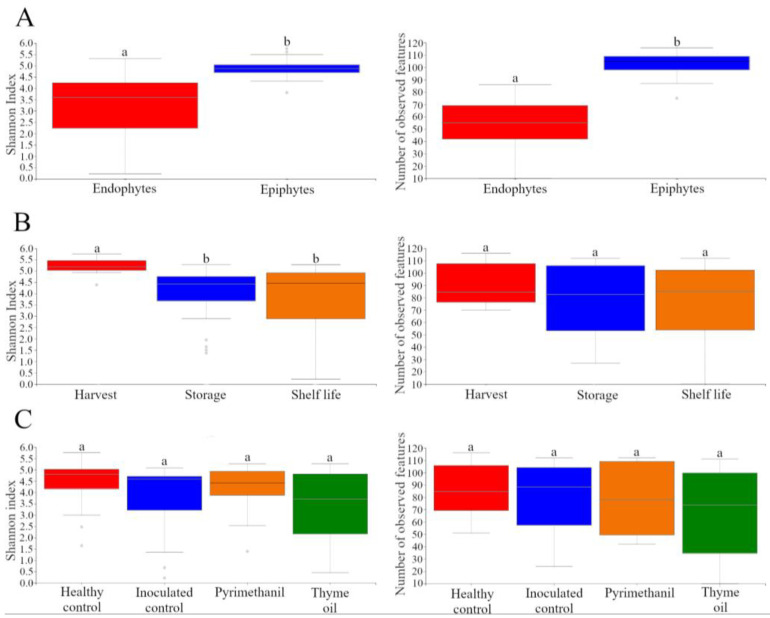
Box and whisker plot for the values of the Shannon index (**left**) and number of observed features (**right**), with samples grouped according to tissue (**A**), time point (**B**), and treatment (**C**). Upper and lower box bounds are associated with the 75th and 25th percentile of the distribution, while upper and lower whisker are associated with the 91st and 9th percentile of the distribution, respectively. For each metric, different letters indicate statistically significant differences between groups (*p* < 0.05).

Figure 2A–C show the Shannon Index and number of observed features values based on tissue, time point, and treatment, respectively. Statistically significant differences between tissues are present for both considered metrics, with higher values for epiphytic samples compared to endophytic samples. For a time point, there are no statistically significant differences in the number of observed features, while the Shannon index shows statistically lower values for storage and shelf-life, compared to harvest. Finally, there are no statistically significant differences for both considered metrics when treatment is taken into account.

**Figure 3:**
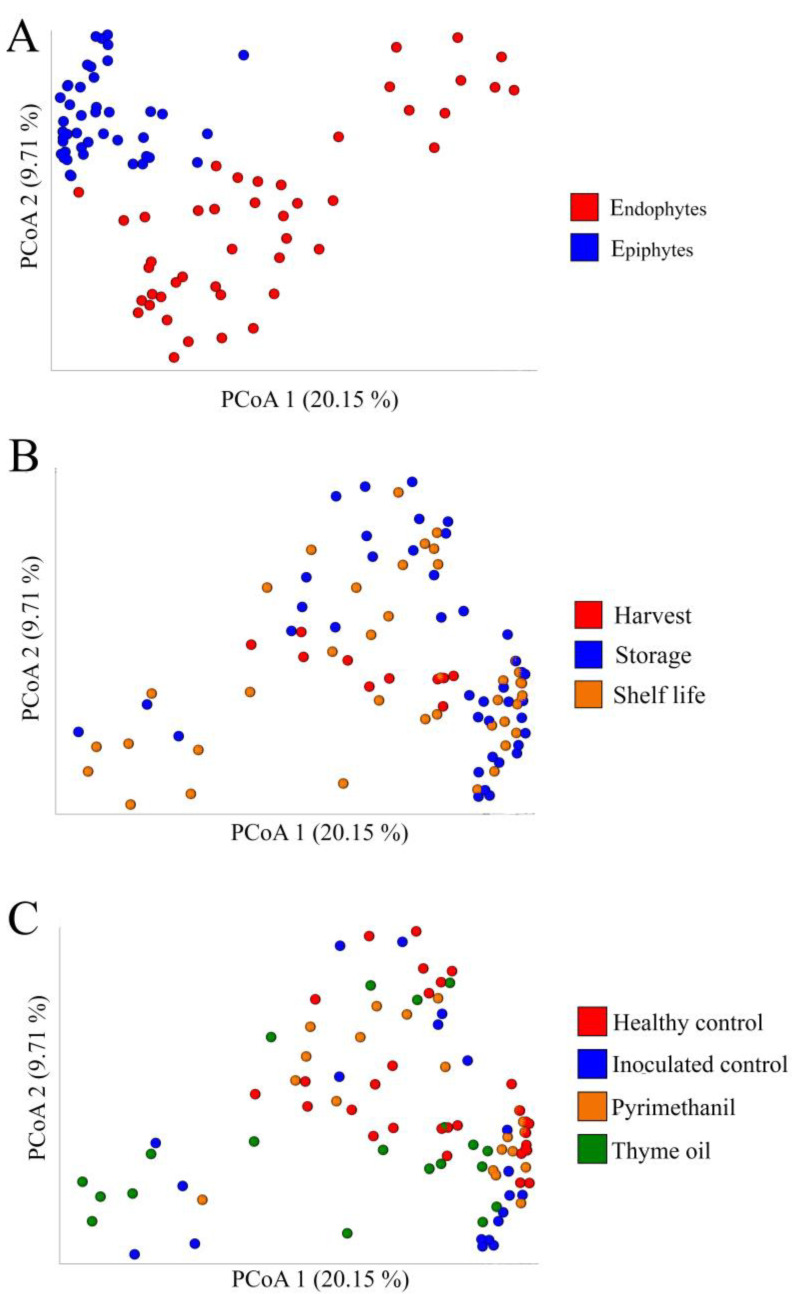
PCoA analysis of Bray-Curtis dissimilarity index results, based on tissue (**A**), time point (**B**), and treatment (**C**).

Similarly, Figure 3A–C are Principal Coordinates Analysis (PCoA) plots based on Bray-Curtis dissimilarity index values for tissue, time point, and treatment, respectively. Samples from different tissues cluster apart from each other, while samples from different treatments and different time points cluster together.

PERMANOVA results on the same data are shown in Table 7. Tissue is the factor associated with the highest explained variance (13%), followed by the sampling time point (8%) and treatment (6.5%). Overall, considered parameters and their interactions are shown to explain little more than 40% of the total variance.

**Figure 4:**
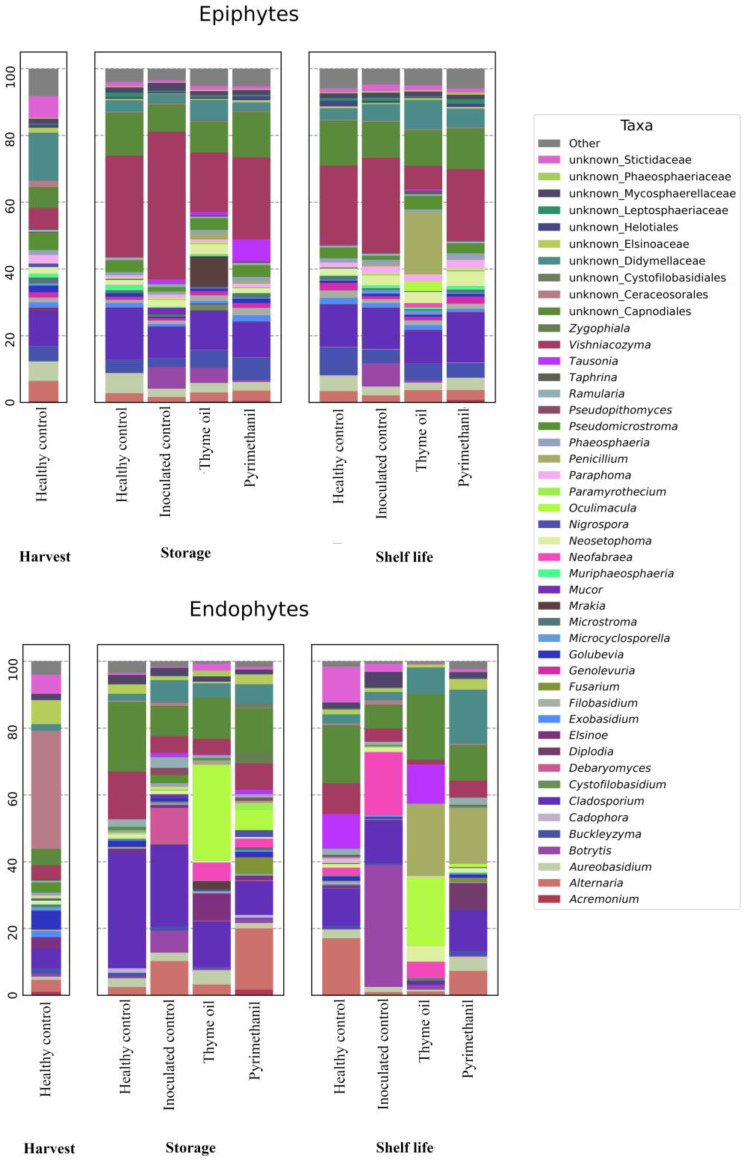
Taxa composition of epiphytic and endophytic fungal communities. The “Other” category includes all taxa with less than 1% relative presence in all considered groups.

Composition data for epiphytic and endophytic samples are shown in Figure 4**.** In endophytic populations, the most abundant clade at harvest consists of unknown Ceraceosorales (35%), which are not encountered in the subsequent time points. In samples inoculated with *Botrytis cinerea,* the genus *Botrytis* showed an appreciable presence after storage (6.5%) and shelf-life (36.5%) only in the absence of treatment. The genus *Oculimacula* is the most abundant (28.5%) clade in inoculated samples treated with thyme oil at the end of storage, whereas it is the second most abundant (20.6%) clade after shelf-life, just behind the genus *Penicillium* (21%). Inoculated samples treated with pyrimethanil have *Alternaria* as the predominant genus (18.3%) during storage, while during shelf-life the genus *Penicillium* becomes predominant (16.5%). The genera *Alternaria, Cladosporium*, and *Vishniacozyma* appear in all samples, albeit with a variable presence. Other notable genera include *Fusarium*, which has a significant presence (4.9%) during storage in inoculated samples treated with pyrimethanil, and *Neofabraea,* which has a significant presence in particular in inoculated control samples after shelf-life (19%).

In epiphytic populations, the taxonomical composition appears more diverse compared to the endophytic samples. Yeast genus *Vishniacozyma* is the main component in all storage and shelf-life groups, with the exception of samples inoculated with *B. cinerea* and treated with thyme oil. *Botrytis* development during storage can be observed in inoculated samples, both untreated (6.5%) and treated with thyme oil (4.5%), while it is almost absent in chemically treated samples (0.6%). In shelf-life samples, *Botrytis* species maintain a similar presence in untreated samples (7%), but not in thyme-oil treated samples, where *Penicillium* becomes the main genus (18.6%).

## 4. Discussion

We assayed the effect of biofumigation with EOs against *Botrytis cinerea* in vitro and in vivo. All three EOs (thyme, savoury, and basil) and the thymol formulation used at a concentration of 1% and thyme and savoury EOs at 0.5%, were effective in inhibiting the growth of *B. cinerea* in vitro. Similarly, in a previous study [41], the EO of *Ocinum basilicum* and *Thymus kotschyanus*, used by spray application showed a high inhibitory effect on the mycelial growth of *B. cinerea* and *p. expansum* in pears. Our results are in agreement with other studies about the use of biofumigation with thyme and savoury EOs in the control of different pathogens including *B. cinerea* and *Colletotrichum gloeosporioides* [17]. The use of thyme EO applied by direct contact against *B. cinerea* and *Penicillium* species provided a complete inhibition of the growth of both pathogens [42,43]. The antifungal activity of essential oils of *Thymus* species has therefore been reported as suitable for applications in the food industry [41,44,45].

Given the efficacy of EOs observed in vitro, whether similar efficacy could be obtained by fumigation with EOs of cold-stored apples was investigated. EO of thyme, savoury, and thymol formulation used at 1% concentration showed significant grey mould control after 60 days of storage at 1 ± 1 °C. The efficacy of biofumigation with thyme and savoury EOs was previously reported on apples cv. Red Fuji [8]. Thyme and savoury EOs, and their major components thymol and carvacrol, previously showed antifungal activity against *B. cinerea* [8,46]. These oils and their main components also controlled other pathogens, such as *Monilinia fructicola* on apricots and plums [47], or peaches and nectarines [11], and *p. expansum* on pears [41]. Essential oils of *Thymus* species and *Mentha* species and their components had much higher antifungal activity than the fungicide bifonazole, as shown in previous studies [20]. In our experiment, the use of 1% basil EO was not effective in controlling grey mould of apples at the end of storage, unlike previous studies [48,49] that showed that spray application of basil EO was effective on apples.

To assess the impact of treatments on apple quality, analyses of firmness, total soluble solids, and titratable acidity parameters were performed. Apples treated with 1% thyme EO were firmer than the control after 30 days of storage at 1 ± 1 °C. A statistically significant difference was observed for apples treated with 1% thyme and 1% savoury EOs, compared to the values obtained for the control after 60 of storage at 1 ± 1 °C. These results are in partial agreement with what was observed on pears treated with thyme, basil, and rosemary EOs by spraying, where no effects were observed on weight loss, pH, and total soluble solids, while differences were reported on taste and firmness [41]. The firmness was reduced by *Thymus kotschyanus* (thyme) EO, whereas basil (*O. basilicum*) EO slowed the softening process. A decrease in firmness is likewise reported by spraying celery, basil, and rosemary EOs [49]. It should be emphasised that in our experiments EOs were applied by biofumigation, as in Santoro et al. [11], who demonstrated that thyme and savoury EO vapours on peaches and nectarines did not influence the fruit quality, but in fact showed a positive effect in reducing weight loss.

During in vivo experiments, sampling of the atmosphere inside the cabinets was performed to characterize and quantify the volatile components of the oils released through biofumigation. Analysis of the chromatographic profiles showed that thymol and carvacrol, which have antimicrobial activity against several pathogens [44,45,50,51], were present in thyme EO. The existence of different taxonomic chemotypes of *Thymus* essential oils should be highlighted, as they may have different biological properties: antifungal, antioxidant, and antimicrobial [41,44,45,50,52]. Though *p*-cymene was one of the main components of thyme EO, it does not have specific antimicrobial properties [53]. Previous studies on the constituents of thyme EOs showed that carvacrol has the highest antimicrobial and antifungal activity [20,44]. The high effectiveness of carvacrol in reducing the growth of *B. cinerea* in vitro and in vivo in grapes was demonstrated by Martínez-Romero and colleagues [51]. The antifungal activity of the thyme EO tested was mainly given by thymol, as carvacrol was present at a very low percentage, throughout the storage and shelf life of the apples.

Carvacrol, thymol, terpinen-4-ol, and linalool determine the antifungal activity in savoury EO [54]. In our savoury EO, carvacrol and *p*-cymene were the main components, whereas Santoro and colleagues [11] who also tested the efficacy of savoury EO, showed that the main compounds detected were linalool and carvacrol.

The gas chromatographic analysis showed the basil EO releases mainly linalool [41]. Linalool, along with estragole, determines the antimicrobial properties of basil EO [55]. Although the percentages of linalool and estragole present in the basil EO were high—15.34% and 57.79%, respectively—the treatments carried out in our experiments were ineffective.

As for the thymol formulation, a reduction of thymol abundance was observed as time increased. The decrease of thymol also permitted maintenance of a certain level of efficacy of the treatment against grey mould in shelf-life, though the apples were no longer in a closed environment.

In addition to EOs’ efficacy against *B. cinerea*, the effect on the fungal microbiome composition was also investigated. Fruits treated with thyme oil were compared to both fruits treated with pyrimethanil and control fruits. By considering sampling time point, treatment, and tissue, no parameter or combination of parameters showed a predominant role in explaining total variance. Tissue had the highest influence among all considered factors, which is mirrored by a clean compositional clustering of epiphytic and endophytic samples. Epiphytic samples showed both higher richness and higher evenness compared to their endophytic counterparts. Host plants were shown actively shaping the endophytic communities of their fruits [56], which, combined with the low oxygen levels [57] and higher sugar content [58] of the subepidermal region, could result in the selection of smaller fungal populations.

Regarding the time point, harvest samples were compositionally different from both storage and shelf-life samples, which is in accordance with previous studies [59,60]. Based on alpha diversity analyses, harvest samples had higher evenness compared to storage and shelf-life samples, but shared the same richness, indicating that—in a context of similar number of taxa-some taxa start to prevail in storage and later in shelf-life. This dynamic has been observed in other studies, as a result of both storage time and temperature [61]. In contrast to both tissue and time point, the treatments did not show any effect on sample composition and did not seem to affect either sample richness or evenness. Despite this, compositional data of our samples do suggest that treatments affect at least some postharvest rot genera. In particular, as observed by Banani and colleagues [8], treatments with thyme oil and a chemical fungicide also result in a lower abundance of *B. cinerea* compared to untreated samples. At the same time, *Penicillium* species was not negatively affected by thyme oil or chemical fungicide treatments, but on the contrary, it increased. *Penicillium* species Was previously demonstrated to be tolerant to thyme oil treatments [20] and some strains are resistant to fungicides [62]. It is thus reasonable to assume that in the presence of these treatments, *Penicillium* species grew by occupying the ecological niche left by other fungal genera.

## 5. Conclusions

Essential oils of thyme and savoury and the thymol formulation are promising for the control of postharvest diseases in apples, showing potential for developing low environmental impact fungicides to be used in sustainable agriculture. We can hypothesize a similar efficacy obtained for ’Opal’ on other apple cultivars. It could be interesting to test different concentrations of oils and to verify the continuous effectiveness of the oils assuming a replacement of the Petri dishes after 30 days of storage to keep the level of volatile organic compounds with antifungal activity. Moreover, we would also like to test the effect of EO biofumigation over longer periods of time, in order to more closely match commercial practises, where apple fruit can remain in cold storage up to 9 months. The development of a slow-release formulation of essential oils should be targeted to favour a constant control of fungal diseases.

## Figures and Tables

**Table 1:** Effect of volatiles released by EOs on mycelial growth (diameter, cm) of two strains of *B. cinerea* in sandwich plate experiments.

Treatment	Concentration	*B. cinerea* BOT1	*B. cinerea* BOT2
		Mean Diameter (mm) ± SD	
Thyme	1%	0.0 ± 0.0 ^a^	0.0 ± 0.0 ^a^
	0.5%	0.0 ± 0.0 ^a^	0.0 ± 0.0 ^a^
	0.1%	8.8 ± 0.4 ^bc^	8.1 ± 0.5 ^b^
Basil	1%	0.0 ± 0.0 ^a^	0.0 ± 0.0 ^a^
	0.5%	2.0 ± 1.1 ^a^	0.0 ± 0.0 ^a^
	0.1%	65.5± 1.8 ^f^	76.3 ± 0.4 ^e^
Savoury	1%	0.0 ± 0.0 ^a^	0.0 ± 0.0 ^a^
	0.5%	0.0 ± 0.0 ^a^	0.0 ± 0.0 ^a^
	0.1%	16.6 ± 0.2 ^d^	14.1 ± 0.5 ^c^
Thymol formulation	1%	2.4 ± 0.2 ^ab^	0.0 ± 0.0 ^a^
	0.5%	10.3 ± 1.1 ^cd^	8.5 ± 0.4 ^bc^
	0.1%	52.6 ± 1.6 ^e^	57.3 ± 1.1 ^d^
Control	Not appliable	77.0 ± 0.0 ^g^	77.0 ± 0.0 ^e^

EOs were applied at different concentrations (0.1, 0.5, and 1.0%). Petri dishes were grown at 20 °C ± 1 °C for 5 days. Different letters (a–g) indicated a significant difference determined by Tukey’s multiple comparison test (*p* < 0.05). Values are indicated as mean ± standard deviation (SD).

**Table 2:** Firmness, total soluble solids, and titratable acidity of apples ‘Opal’ treated with essential oil biofumigation and stored at 1 ± 1 °C with 95% relative humidity for 60 days.

Time Point	Treatment	Firmness [N/cm^2^] ± SD *	Total Soluble Solids (TSS) [%] ± SD *	Titratable Acidity [%] ± SD *
At harvest		43.91 ± 4.01	14.09 ± 1.19	0.60 ± 0.06
30 days	Thymol formulation (1%)	39.33 ± 2.84 ^ab^	15.03 ± 0.82 ^a^	0.42 ± 0.02 ^a^
	Thyme (1%)	40.83 ± 4.52 ^b^	15.32 ± 0.85 ^a^	0.44 ± 0.04 ^a^
	Savoury (1%)	38.06 ± 3.13 ^ab^	15.24 ± 1.25 ^a^	0.45 ± 0.09 ^a^
	Basil (1%)	38.38 ± 1.98 ^ab^	15.30 ± 1.25 ^a^	0.40 ± 0.12 ^a^
	Control	34.40 ± 0.88 ^a^	14.54 ± 0.41 ^a^	0.40 ± 0.03 ^a^
60 days	Thymol formulation (1%)	34.30 ± 2.66 ^a^	15.12 ± 0.83 ^a^	0.36 ± 0.02 ^a^
	Thyme (1%)	38.17 ± 7.66 ^a^	15.33 ± 0.98 ^a^	0.42 ± 0.04 ^b^
	Savoury (1%)	34.79 ± 5.10 ^a^	15.38 ± 0.85 ^a^	0.43 ± 0.01 ^c^
	Basil (1%)	34.08 ± 4.31 ^a^	15.59 ± 1.36 ^a^	0.35 ± 0.03 ^a^
	Control	30.58 ± 4.12 ^a^	15.09 ± 0.89 ^a^	0.36 ± 0.03 ^a^

Each value of firmness, total soluble solids, and titratable acidity is the mean of n = 3 replicates with five fruits. Values at the same time point, followed by the same letter, are not statistically different in acoordance with Tukey’s test (*p* < 0.05). * Values are expressed as a mean of five replicates ± standard deviation (SD).

**Table 3:** Composition of thymol formulation and characterization of the atmosphere of cabinet during the storage trial on apples ‘Opal’ obtained by GC-MS and SPME-GC-MS analysis, respectively. Air in the cabinet was sampled at 1, 30, and 60 days of storage.

VOCs	EO Composition (%)	Days of Storage		
		1 Day	30 Days	60 Days
		ppm ± SD *		
Thymol	18.81	4.02 ± 0.23	1.39 ± 0.05	0.64 ±0.03
Propylene glycol	6.87			
Other compounds	74.32			

* Values are expressed as a mean of five replicates ± standard deviation (SD).

**Table 4:** Composition of thyme essential oil and characterization of the atmosphere of cabinet during the storage trial on apples ‘Opal’ obtained by GC-MS and SPME-GC-MS analysis, respectively. Air in the cabinet was sampled at 1, 30, and 60 days of storage.

VOCs	EO Composition (%)	Days of Storage		
		1 Day	30 Days	60 Days
		ppm ± SD *		
Borneol	1.77	0.00 ± 0.00	1.08 ± 0.19	0.20 ± 0.00
Camphene	0.72	0.00 ± 0.00	0.00 ± 0.00	0.00 ± 0.00
β-Caryophyllene	1.46	4.21 ± 0.52	0.31± 0.12	0.31 ± 0.11
Carvacrol	4.77	0.57 ± 0.18	0.27 ± 0.13	0.21 ± 0.01
Eucalyptol	0.86	0.00 ± 0.00	0.00 ± 0.00	0.00 ± 0.00
Limonene	0.56	0.00 ± 0.00	0.00 ± 0.00	0.00 ± 0.00
Linalool	6.42	2.06 ± 0.12	0.80 ± 0.01	0.00 ± 0.00
O-Methylthymol	0.43	0.00 ± 0.00	0.39 ± 0.10	0.41 ± 0.07
β-Myrcene	1.59	0.00 ± 0.00	0.50 ± 0.05	0.00 ± 0.00
α-Pinene	1.89	1.30 ± 0.01	1.05± 0.02	0.00 ± 0.00
β-Pinene	1.77	1.77 ± 0.01	1.16 ± 0.06	0.00 ± 0.00
*p*-Cymene	20.39	11.40 ± 1.82	26.24 ± 4.90	2.61 ± 0.18
γ-Terpinene	6.64	6.16 ± 1.03	2.59 ± 0.09	0.38 ± 0.06
Terpinen-4-olo	0.64	0.00 ± 0.00	0.00 ± 0.00	0.00 ± 0.00
α-Terpineol	0.50	0.00 ± 0.00	0.00 ± 0.00	0.00 ± 0.00
α-Terpinolene	1.47	0.00 ± 0.00	0.00 ± 0.00	0.00 ± 0.00
**Thymol**	45.95	13.38 ± 2.20	5.13 ± 0.32	3.85 ± 0.57
α-Thujene	0.56	0.00 ± 0.00	0.00 ±0.00	0.00 ± 0.00
Other compounds	1.61	0.00 ± 0.00	0.00 ± 0.00	0.00 ± 0.00

* Values are expressed as a mean of five replicates ± standard deviation (SD).

**Table 5:** Composition of savoury essential oil and characterization of the atmosphere of cabinet during the storage trial on apples ‘Opal’ obtained by GC-MS and SPME-GC-MS analysis, respectively. Air in the cabinet was sampled at 1, 30, and 60 days of storage.

VOCs	EO Composition (%)	Days of Storage		
		1 Day	30 Days	60 Days
		ppm ± SD *		
Borneol	0.74	0.00 ± 0.00	1.60 ± 0.03	0.00 ± 0.00
β-Caryophyllene	1.20	0.00 ± 0.00	0.00 ± 0.00	0.00 ± 0.00
**Carvacrol**	60.33	8.64 ± 0.27	34.28 ± 3.90	3.34 ± 0.30
*p*-Cymene	17.07	4.69 ± 0.91	25.04 ± 2.76	3.95 ± 0.56
β-Myrcene	1.70	0.27 ± 0.04	0.90 ± 0.08	0.16 ± 0.01
α-Pinene	1.40	0.03 ± 0.00	0.37 ± 0.07	0.00 ± 0.00
β-Pinene	0.60	0.00 ± 0.00	0.00 ± 0.00	0.00 ± 0.00
γ-Terpinene	6.80	5.34 ± 0.42	5.61 ± 0.24	0.90 ± 0.07
Terpinen-4-ol	1.26	0.00 ± 0.00	2.27 ± 0.74	0.26 ± 0.01
α-Terpineol	0.60	0.00 ± 0.00	0.00 ± 0.00	0.00 ± 0.00
Terpinolene	0.72	0.00 ± 0.00	0.00 ± 0.00	0.00 ± 0.00
α-Thujene	0.66	0.00 ± 0.00	0.06 ± 0.00	0.00 ± 0.00
Thymol	5.52	0.31 ± 0.02	1.98 ± 0.13	0.24 ± 0.02
Other compounds	1.40	0.00 ± 0.00	0.00 ± 0.00	0.00 ± 0.00

* Values are expressed as a mean of five replicates ± standard deviation (SD).

**Table 6:** Composition of basil essential oil and characterization of the atmosphere of cabinet during the storage trial on apples ‘Opal’ obtained by GC-MS and SPME-GC-MS analysis, respectively. Air in the cabinet was sampled at 1, 30, and 60 days of storage.

VOCs	EO Composition (%)	Days of Storage		
		1 Day	30 Days	60 Days
		ppm ± SD *		
*p*-Anisaldehyde	2.68	0.88 ± 0.02	1.01 ± 0.17	0.49 ± 0.03
t-α-Bergamotene	0.47	6.60 ± 8.63	1.11 ± 0.08	0.49 ± 0.03
α-Bisabolene	1.15	0.00 ± 0.00	0.59 ± 0.06	0.00 ± 0.00
β-Bisabolene	1.23	0.00 ± 0.00	0.27 ± 0.20	0.00 ± 0.00
Borneol	0.54	0.00 ± 0.00	0.00 ±0.00	0.00 ± 0.00
β-Cedrene	0.28	0.00 ± 0.00	0.00 ± 0.00	0.00 ± 0.00
α-Citral	0.16	0.21 ± 0.00	0.00 ± 0.00	0.00 ± 0.00
β-Citral	0.27	0.00 ± 0.00	0.00 ± 0.00	0.00 ± 0.00
**Estragole**	57.79	128.60 ± 5.53	32.07 ± 1.98	31.42 ± 4.04
α-Farnesene	5.03	24.68 ± 0.34	111.44 ± 14.78	31.15 ± 3.82
Fenchone	5.21	0.00 ± 0.00	0.00 ± 0.00	0.00 ± 0.00
α-Humulene	0.13	0.45 ± 0.06	0.17 ± 0.02	0.48 ± 0.09
Linalool	15.34	57.76 ± 4.36	2.68 ± 3.05	0.66 ± 0.05
cis-Linanool oxide	5.40	0.00 ± 0.00	0.00 ± 0.00	0.36 ± 0.05
Menthol	0.40	0.00 ± 0.00	0.00 ± 0.00	0.00 ± 0.00
Terpinen-4-ol	0.66	0.00 ± 0.00	0.00 ± 0.00	0.00 ± 0.00
α-Terpineol	0.88	0.00 ± 0.00	0.00 ± 0.00	0.00 ± 0.00
Other compounds	2.38	0.00 ± 0.00	0.00 ± 0.00	0.00 ± 0.00

* Values are expressed as a mean of five replicates ± standard deviation (SD).

**Table 7:** PERMANOVA analysis results for fungi. The R2 value indicates the total variance fraction explained by the parameter or combination of parameters, while Pr (>F) is the FDR-adjusted *p*-value using the Benjamini-Hochberg method. For significance levels, ** = *p* < 0.01, *** = *p* < 0.001, and n.s. = not significant.

Parameter	R2	Pr (>F)
Sampling time point	0.081	***
Treatment	0.066	***
Tissue	0.135	***
*Botrytis* inoculum	0.029	***
Sampling time point × Treatment	0.030	**
Sampling time point × *Botrytis* inoculum	0.009	n.s.
Sampling time point × Tissue	0.040	**
Treatment × Tissue	0.035	***
*Botrytis* inoculum × Tissue	0.009	n.s.
Sampling time point × Treatment × Tissue	0.016	n.s.
Sampling time point × *Botrytis* inoculum × Tissue	0.008	n.s.
Residuals	0.541	/
**Total**	**1.000**	**/**
